# Transcranial Direct Current Stimulation Over the Right Temporal Parietal Junction Facilitates Spontaneous Micro-Expression Recognition

**DOI:** 10.3389/fnhum.2022.933831

**Published:** 2022-07-08

**Authors:** Yue Ge, Rui Su, Zilu Liang, Jing Luo, Suizi Tian, Xunbing Shen, Haiyan Wu, Chao Liu

**Affiliations:** ^1^State Key Laboratory of Cognitive Neuroscience and Learning and IDG/McGovern Institute for Brain Research, Beijing Normal University, Beijing, China; ^2^Center for Collaboration and Innovation in Brain and Learning Sciences, Beijing Normal University, Beijing, China; ^3^Beijing Key Laboratory of Brain Imaging and Connectomics, Beijing Normal University, Beijing, China; ^4^Beijing Institute of Biomedicine, Beijing, China; ^5^School of Psychology, Beijing Normal University, Beijing, China; ^6^College of Humanities, Jiangxi University of Chinese Medicine, Nanchang, China; ^7^Centre for Cognitive and Brain Sciences and Department of Psychology, University of Macau, Taipa, China

**Keywords:** transcranial direct current stimulation, right temporal parietal junction, micro-expression training, artificial micro-expression, spontaneous micro-expression

## Abstract

Micro-expressions are fleeting and subtle emotional expressions. As they are spontaneous and uncontrollable by one’s mind, micro-expressions are considered an indicator of genuine emotions. Their accurate recognition and interpretation promote interpersonal interaction and social communication. Therefore, enhancing the ability to recognize micro-expressions has captured much attention. In the current study, we investigated the effects of training on micro-expression recognition with a Chinese version of the Micro-Expression Training Tool (METT). Our goal was to confirm whether the recognition accuracy of spontaneous micro-expressions could be improved through training and brain stimulation. Since the right temporal parietal junction (rTPJ) has been shown to be involved in the explicit process of facial emotion recognition, we hypothesized that the rTPJ would play a role in facilitating the recognition of micro-expressions. The results showed that anodal transcranial direct-current stimulation (tDCS) of the rTPJ indeed improved the recognition of spontaneous micro-expressions, especially for those associated with fear. The improved accuracy of recognizing fear spontaneous micro-expressions was positively correlated with personal distress in the anodal group but not in the sham group. Our study supports that the combined use of tDCS and METT can be a viable way to train and enhance micro-expression recognition.

## Introduction

The capacity to use emotional signals is vital for navigation in daily life. Identifying rapidly changing facial expressions to monitor others’ intentions and adjusting reactions accordingly is crucial for establishing, developing and maintaining stable interpersonal relationships (Schönenberg et al., 2014; Alguacil et al., 2017; Hubble et al., 2017). Both macro-expressions and micro-expressions exist in human interactions. Macro-expressions, the full facial expression, lasting from 1/2 s to 4 s, are typical and common in daily life. Less often, when people attempt to conceal or repress their emotions, a short micro-expression (usually lasting from 1/25 s to 1/5 s, up to 1/2 s) is embedded in the flow of expressions. Since micro-expressions are automatic and uncontrollable, they are considered to reveal the genuine feelings that individuals attempt to conceal. The authenticity and objectivity of micro-expression recognition processes make them of great value in diverse fields such as forensic investigation, clinical diagnosis, and national security (Ekman, 2009a; Frank et al., 2009; Weinberger, 2010). Likewise, the ability to “read” others’ true emotional states accurately is also advantageous for ordinary people and has been linked to social communication skills. For instance, individuals with higher micro-expression reading accuracy received better evaluations from their supervisors at the workplace (Matsumoto and Hwang, 2011). However, since micro-expressions are very subtle and rapid, recognizing them in real life is a practical challenge (Ekman et al., 1987; Shen et al., 2016).

The Micro-Expression Training Tool (METT) is a definitive training tool used to enhance the micro-expression recognition ability. Previous research has indicated that the METT can significantly increase the accuracy of recognizing micro-expressions. Using METT for less than 40 min improved a respondent’s recognition accuracy by 30–40% (Ekman, 2009b). It is noteworthy that the micro-expression material used in Ekman’s METT for test and practice is artificial dynamic expression made from three consecutive facial images (Matsumoto et al., 2000). However, in real human interactions, spontaneous micro-expression is not only a dynamic but also a continuously changing expression with a short duration and subtle amplitude. Given the vast differences between artificial and spontaneous micro-expressions, the ideal training tool for micro-expression recognition would use spontaneous micro-expression stimuli to achieve good ecological validity. Therefore, the METT used in the present study to assess the training effect included artificial and spontaneous micro-expressions (Chen et al., 2018). Specifically, artificial micro-expressions were selected from POFA, NimStim, and TFEID (Ekman, 1993; Chen and Yen, 2007; Yan et al., 2014, 2013). Spontaneous micro-expressions were real facial micro-expression videos selected from the Chinese Academy of Sciences Micro-Expression (CASME) database (Yan et al., 2014).

Facial expression recognition consists of the implicit perception and explicit recognition processes. Implicit facial expression perception, occurring relatively quickly, can be made with simple and familiar emotional information input and without consciousness. While explicit facial expression recognition is a process of inferring the emotional state based on facial expression cues, and requires comparison between the currently obtained features and related prior knowledge (Wicker et al., 2008; Rellecke et al., 2012; Xiao et al., 2016). In the training section as main part of the METT, individuals can learn how to read other people’s emotions accurately based on facial information. Specifically, a narrator describes unique facial cues to signal emotions and provides explicit examples of dynamic changes in the regions of the eyes, nose, and mouth. An example is the micro-expression of anger in which—”the mouth is sometimes open and sometimes closed, whereas the eyebrows are always pulled down and close to the inside, the upper eyelids deepen, and the lower eyelids shrink, causing the eyes to glare widely.” (Ekman, 2003). According to the different functions of the implicit and explicit facial processes, this method of describing the unique features of various categories of expressions to help trainees better distinguish emotional expressions mainly works on the explicit route of facial expression recognition (Ekman, 2003; Endres and Laidlaw, 2009). Therefore, presumably, the training effect can be enhanced by improving the explicit recognition ability. Existing neuroimaging studies indicated that regions overlapping with the inferior portion of the temporal parietal junction (TPJ) were linked to the identification of facial expressions (Carter and Huettel, 2013). Critchley et al. (2000) investigated the two parallel pathways of facial expression recognition and found that explicit processing activated the temporal cortex while implicit processing evoked the amygdala. Strong structural and functional links between amygdala and temporal parietal regions have been identified (Bickart et al., 2014). A study in macaques revealed that electrical microstimulation of a face-processing region of the temporal lobe activated the amygdala (Moeller et al., 2008). Amygdala played a central role in processing unconscious stimuli through a subcortical route and was implicated in coarse processing of emotion. TPJ was responsible for the attention reorienting/integration and further processing emotional information deeply (Dzafic et al., 2019). Monk et al. (2010) found that when attention bias to emotional faces was equivalent, autism participants had weak connectivity between amygdala-temporal lobe during emotional face processing, which may imply their obstructions to transmission in emotion processing. Olsson and Ochsner (2008) analyzed the role of social cognition in emotion and suggested that the right TPJ (rTPJ) interconnected with visuospatial centers supported externally generated representations and might code cognitive aspects in this way. Qiao et al. (2020) found that anodal HD-tDCS over the rTPJ significantly increased fixation time and fixation count in emotional cue area and better utilized facial expression information to infer emotional states. Meanwhile, other studies further support the involvement of the rTPJ in the explicit facial expression recognition (Kesler-West et al., 2001; Wicker et al., 2008; Atique et al., 2011).

Transcranial direct current stimulation (tDCS) is a non-invasive neuromodulation technique involving the delivery of small direct currents through two surface electrodes on the scalp to modulate cortical excitability in the underlying brain region. The excitability can be increased by anodal stimulation and decreased by cathodal stimulation (Hogeveen et al., 2014; Chrysikou et al., 2017). In this study, we administered the anodal stimulation over the rTPJ and expected to observe an improvement in explicit expression recognition ability — reflected in micro-expression training and measured through the improved accuracy of micro-expression recognition. Specifically, we assumed that the anodal group would demonstrate higher scores after training compared to the sham group. In addition, compared with the artificial micro-expressions synthesized from three static expressions, spontaneous micro-expressions from the real facial micro-expression video depicted a new and unique expression at each time point, and therefore provided more expression information (social information). When dealing with these more complex, natural, and ecologically valid stimuli, a higher level of social cognitive function is required. As the rTPJ was sensitive to processing social information (Tang et al., 2015; Kelley et al., 2021), it is likely to observe a better training effect on spontaneous micro-expression recognition with anodal stimulation. Previous research also demonstrated that the rTPJ played a role in attentional orientation toward threat information (Sagliano et al., 2019). One study indicated increased fear-specific activation in the rTPJ (Zwanzger et al., 2014). Donaldson et al. (2019) discovered that anodal rTPJ high-definition tDCS (HD-tDCS) improved the facial emotion processing performance of fear, and the stimulation effects depended on the intensity and salience/valence (negative/threat) of the emotion. Therefore, it is possible that a stronger effect of training on fearful expression recognition is observed after anodal stimulation.

Previous research highlighted the close relationship between emotion recognition and empathy (Riess et al., 2012; Zhang et al., 2017; Yang et al., 2018). Empathy has been defined as the understanding of their emotions and internal thoughts (cognitive empathy) and sharing the emotional state of others (affective empathy) (Bloom, 2017; Israelashvili et al., 2020). Facial emotion recognition overlapped widely with empathy in terms of the perceptual inputs of emotional empathy (i.e., visual facial emotional stimuli) and the inferential processes of cognitive empathy (Holland et al., 2021). A previous study supported that empathy improved the recognition of facial emotion expressions, such as those expressing fear (Besel and Yuille, 2010). Emotion perception was correlated to affective and cognitive empathy with different effect sizes (Olderbak and Wilhelm, 2017). Moreover, Israelashvili et al. (2020) found that two facets of affective empathy had opposite relationships to facial expression recognition: empathic concern and personal distress were positively and negatively correlated, respectively, with accurate emotion recognition. Interestingly, personal distress, the “self-oriented” feeling of personal anxiety and unease in response to extreme distress in others, was detrimental to expression recognition because of its overly self-focused reaction (Davis, 1980, 1983). However, anodal stimulation over the rTPJ could inhibit self-centered concern and promote other-oriented concern (Payne and Tsakiris, 2017; Martin et al., 2019) that may improve facial expression recognition accuracy and thus may enhance the training effect of the individuals with personal distress. Nevertheless, considering the complex relationship among empathy, rTPJ and facial expression recognition, we conducted exploratory analyses to investigate the role of all empathy traits with the Interpersonal Reactivity Index (IRI) questionnaire in the effect of anodal stimulation over the rTPJ on training effect.

In the current study, we aimed to examine whether targeting tDCS over the rTPJ enhanced the effect of training on recognizing artificial and spontaneous micro-expressions using the Chinese version of METT. We also sought to understand the role of empathy in this process. We expected a significant training effect for both artificial and spontaneous micro-expression by using the Chinese version of METT. The anodal stimulation would enhance the effect of training on micro-expression recognition, especially for fear spontaneous ones. In light of prior findings regarding the role of rTPJ in facilitating the transformation of self to other representation, it was also hypothesized that anodal stimulation would enhance the effect of training in individuals with personal distress.

## Materials and Methods

### Participants

Using G*Power 3.1.9.4 software, *a priori* calculation indicated a required minimum sample size of thirty-four participants to detect adequate power (1 – β = 0.8) and medium-sized effect (*f* = 0.25). Fifty-eight healthy, right-handed, college students took part in our study (mean age = 21.71 ± 2.75 years; 47% males, 53% females). We used a brief self-report questionnaire to obtain information on the participants’ gender, age, and history of psychopathological disorders, including seizure, tumor, stroke, mood disorder, brain surgery or intracranial metal implantation. None of the participants reported a history of neurological or psychiatric disorders. No one dropped out of the experiment. Based on the reported partial η^2^ value of 0.07 in the current study that corresponds to an effect size *f* of 0.27, our sample size of 58 participants divided into two groups who each have two measurements would yield an estimated power of 98.25% to detect significant interaction between stimulation and testing stage for spontaneous micro-expression, which was used as the primary outcome measure. All participants signed a written informed consent form and were paid for their participation. This study was approved by the Institutional Review Board of the State Key Laboratory of Cognitive Neuroscience and Learning at Beijing Normal University.

### Procedure

**FIGURE 1:**
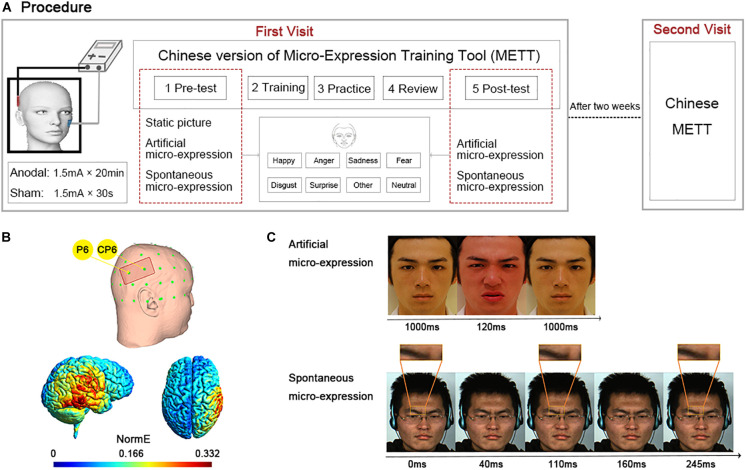
Experiment design. **(A)** Experimental procedure. Two visits were made. In the first visit, participants underwent anodal or sham stimulation and then completed the Chinese version of Micro-Expression Training Tool (METT); in the second visit 2 weeks later, they only finished the Chinese version of METT. The Chinese version of METT included five sections, pre-test, training, practice, review, and post-test. In the sections of pre-test and post-test, participants were asked to choose one of eight emotion labels after seeing the stimuli. The stimuli of pre-test included static expressions, artificial and spontaneous micro-expressions. The stimuli of post-test included artificial and spontaneous micro-expressions. **(B)** Placement of the anodal electrode for the right temporal parietal junction (rTPJ) between P6 and CP6 regions (top row) and the normalized electric field (NormE) derived from electric field modeling calculations using SimNIBS (bottom row). **(C)** The time series of artificial and spontaneous micro-expression in disgust. Source: L.F. Chen and Y.S. Yen, Taiwanese facial expression image database, Brain Mapping Laboratory, Institute of Brain Science, National Yang-Ming University, 2007.

All participants made two visits (see Figure 1A). During the first visit, they received different types of tDCS stimulation depending on whether they belonged to the anodal or the sham group. Participants then completed the Chinese version of METT and filled in the IRI questionnaire. Two weeks later, during the second visit, they performed only the Chinese version of METT. Participants were randomly assigned to the anodal (*n* = 30; 47% males, 53% females) or the sham (*n* = 28; 46% males, 54% females) group. The group assignment was double-blind by using the “study mode” feature of the DC-Stimulator Plus to ensure that neither the experimenters nor the participants were aware of the actual stimulation being administered.

### The Chinese Version of Micro-Expression Training Tool

The Chinese version of METT expands upon the Ekman METT also includes five sections: pre-test, training, practice, review, and post-test. In the pre-test section, participants’ baseline ability of facial expression recognition was measured. The test materials included 84 static expressions with seven basic emotions (twelve each of anger, disgust, fear, happiness, sadness, surprise, and neutral), 14 artificial micro-expressions selected from POFA (Ekman, 1993) (Caucasian models), NimStim (black models), and TFEID (Chen and Yen, 2007) (Asian models) and with the same seven emotions (two each of anger, disgust, fear, happiness, sadness, surprise and neutral), as well as 9 spontaneous micro-expressions, real facial micro-expression videos, selected from the CASME database (Yan et al., 2013, 2014) with five emotions (two each of disgust, happiness, sadness and surprise, as well as one of fear).

The static expression was presented until participants responded. The artificial micro-expression consisted of three successive facial pictures. The target facial picture was sandwiched between two neutral pictures for 120ms. The spontaneous micro-expression lasted more than 5000 ms, but the target spontaneous micro-expression duration was less than 250 ms (1/4 s). To illustrate, the artificial and spontaneous micro-expressions for disgust were highlighted separately in Figure 1C. All materials were presented in random order and balanced for expresser gender, ethnicity, and emotional dimensions. Participants in the pre-test section were asked to choose a single corresponding emotion from the eight emotional labels including anger, disgust, fear, happiness, sadness, surprise, neutral along with an ‘other’ option to help prevent any artifactual agreement. The accuracy of emotion recognition as a dependent variable for this assessment was computed by coding one when participants matched labels successfully and zero when they selected incorrectly, and was expressed as a percentage.

The next section of the training included several Chinese versions of commentary videos in which the narrator emphasized critical facial features and explained how to recognize and distinguish confusing emotions accurately. In the practice section, participants practiced with feedback to ensure they understood and internalized the knowledge and skills learned in the earlier section. The review section repeated the training section. The final post-test followed the same form as the pre-test, but alternative materials were used to assess the ability of artificial and spontaneous micro-expression recognition after training.

All facial images and videos were presented centrally.

### Transcranial Direct-Current Stimulation Manipulation

A battery-driven constant current stimulator device (DC-Stimulator Plus, NeuroConn GmbH, Germany) was used to induce tDCS and deliver low amplitude direct current to the scalp via two saline-soaked sponge electrodes. In this experiment, a 35 cm^2^ anodal electrode (7 cm × 5 cm) was placed between the CP6 and P6 regions (10–20 EEG system, see Figure 1B), which covered the MNI coordinates [54, –59, 22] of the rTPJ reported in previous fMRI studies (Jurcak et al., 2007). The other reference electrode was placed on the left cheek based on previous reports (Hsu et al., 2011; Reinhart et al., 2016; Shires et al., 2020). For the anodal condition, the stimulation with a current intensity of 1.5 mA was delivered continuously for 20 min, and its fade-in and fade-out time were both 15 s. For the sham condition, all settings were identical to the anodal condition except for the duration of real stimulation. For the sham condition, after continuously delivering the current for 30 s, the current was turned off, but the electrodes were kept on the head. This procedure allowed the sham stimulation to be comparable in sensation to the anodal stimulation (Gandiga et al., 2006). At the end of the experiment, when asked to report their impression of whether they had received a real or sham stimulation, all participants believed that the real stimulation was delivered during the experiment.

Simulation of electric field distributions in the brain for the tDCS (see Figure 1B) was performed using the SimNIBS 3.2 (Thielscher et al., 2015) incorporating the template head model included in the software with the following parameters: 1 mm thick rubber electrodes with rectangular connectors encased in 3 mm thick sponges and default tissue connectivity values (white matter: 0.126 S/m, gray matter: 0.275 S/m, cerebrospinal fluid: 1.654 S/m, bone: 0.010 S/m, scalp: 0.465 S/m).

### Empathy Trait Assessment

The IRI questionnaire (Davis, 1980) includes 28 items to assess empathy on a 5-point Likert scale ranging from 0 (does not describe me well) to 4 (describe me very well). The IRI consists of four subscales—empathic concern (α = 0.80), personal distress (α = 0.75), perspective taking (α = 0.79), and fantasy (α = 0.82) —and has good validity and reliability. In this scale, empathic concern (EC) measures the degree of warmth, compassion, and care for others; personal distress (PD) assesses self-oriented discomfort caused by stressful interpersonal situations or emergencies; perspective taking (PT) reflects the tendency to adopt the psychological perspectives of others spontaneously; and fantasy (FS) taps the tendency to imaginatively transpose oneself into fictional situations (Davis, 1983).

### Statistical Analysis

Descriptive data of the participants from the anodal and sham groups were shown as mean ± SD. Between-group baseline characteristic comparisons were performed using the independent sample *t*-tests for continuous variables and the chi-square tests for categorical variables. To examine the effect of tDCS on micro-expression training, we separately ran a mixed two-way repeated ANOVA on the accuracy of artificial and spontaneous micro-expression recognition, with stimulation (anodal/sham) as a between-subject variable and testing stage (pre-test/post-test) as a within-subject variable. To investigate the impact of tDCS on micro-expression recognition training of different emotions, we conducted a mixed two-way repeated ANOVA on the improved accuracy of the micro-expression recognition, with stimulation as a between-subject variable and emotion (disgust, fear, happiness, sadness and surprise) as a within-subject variable. Moreover, we also conducted five independent sample *t*-tests on the improved accuracy of spontaneous micro-expression recognition between two groups to analyze which emotion training effect can be improved using anodal stimulation on exploratory. To explore how tDCS together with empathy traits (empathic concern, personal distress, perspective taking, and fantasy) contributed to the micro-expression training effect, we tested the relationships between empathy traits and the improved accuracy of micro-expression recognition with significant between-group differences by using Spearman rank correlations in each group. The improved accuracy of the micro-expression recognition was calculated by subtracting pre-test accuracy from post-test accuracy. The statistical significance was set at a two-tailed *t*-test (*p* < 0.05). Bonferroni adjustments were used to reduce the risk of Type I error where multiple statistical tests were conducted. However, our results didn’t survive Bonferroni’s correction for multiple comparisons with the statistical threshold adjusted to 0.05/5 = 0.01 for multiple independent sample *t*-tests and 0.05/10 = 0.005 for multiple Spearman rank correlations. Therefore, we complemented the classical frequentist statistical analyses by Bayesian independent *t*-tests and Bayesian Spearman’s rank correlations in the JASP (version 0.16: JASP Team, 2021) and R (version 3.6.3: R Core Team, 2020; van Doorn et al., 2020). The resulting Bayes factors (BF_10_) reflect the ratio of the extent to which the data support the hypothesis, rather than its complement. Therefore, BF_10_ > 1: more support for the hypothesis than for its complement; BF_10_ ≈ 1: inconclusive; BF_10_ < 1: more support for the complement of the hypothesis.

## Results

Thirty participants (mean age = 21.83 ± 3.35 years, 47% males) and twenty-eight participants (mean age = 21.57 ± 1.95 years, 46% males) were assigned to the anodal and sham groups, respectively. There was no significant difference between the two groups in terms of age [*t*(56) = 0.37, *p* = 0.72, Cohen’s *d* = 0.01] and gender (χ^2^ = 0.0003, *p* = 0.99). Furthermore, we conducted *t*-tests to discern differences between the two groups in baseline characteristics, including empathy traits and primary recognition accuracy of static expressions, artificial micro-expressions and spontaneous micro-expressions, and found no significant differences (*ps* > 0.18). Baseline characteristics and corresponding statistics for the two stimulation groups are represented in Table 1.

**TABLE 1:** Characteristics of anodal and sham groups.

	Anodal (*n* = 30)		Sham (*n* = 28)		Between group comparison	
Variable	*M*	SD	*M*	SD	T	*P*
Age (years)	21.83	3.35	21.57	1.95	0.37	0.72
*Primary recognition accuracy (%)*						
Static expression	71.98	6.81	71.17	7.14	0.44	0.66
Artificial micro-expression	59.76	13.85	54.85	13.51	1.37	0.18
Spontaneous micro-expression	41.11	18.94	40.28	17.28	0.18	0.87
*Empathy traits*						
Perspective taking	2.69	0.59	2.51	0.57	1.21	0.23
Fantasy	2.76	0.62	2.83	0.65	−0.42	0.68
Empathy concern	2.65	0.64	2.55	0.63	0.61	0.55
Personal distress	2.28	0.77	2.53	0.60	−1.37	0.18
Interpersonal reactivity index	2.59	0.48	2.60	0.42	−0.07	0.95

*M = Mean; SD = Standard deviation.*

**FIGURE 2:**
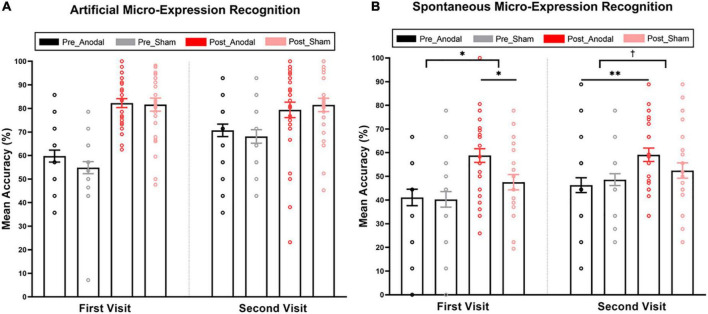
Mean accuracy of artificial and spontaneous micro-expression recognition in pre-test and post-test for the anodal and sham group. **(A)** Mean accuracy of artificial micro-expression recognition. **(B)** Mean accuracy of spontaneous micro-expression recognition. The interaction of stimulation and study was significant in the first visit and marginally significant in the second visit. The anodal group performed better than the sham group after training in the first visit. The anodal group rather than the sham group could improve the accuracy of spontaneous micro-expression recognition through training, even if no stimulation was applied in the second visit. Pre_Anodal and Pre_Sham refer to the mean accuracy of the micro-expression recognition in pre-test for the anodal and sham group. Post_Anodal and Post_Sham refer to the mean accuracy of the micro-expression recognition in post-test for the anodal and sham group. The significance levels are marked by different symbols. “^**^”, “*”, and “†” indicate *p* < 0.01, *p* < 0.05, and *p* < 0.1 (two-tailed), respectively. Error bars represent standard error of mean (SEM).

Repeated ANOVA indicated a training effect on artificial micro-expression recognition. The mean accuracy of artificial micro-expression recognition for each condition is displayed in Figure 2A. The main effect of the testing stage was significant for the first visit [*F*(1,56) = 146.91, *p_*corrected*_* < 0.001, η^2^_*p*_ = 0.72] and second visit [*F*(1,56) = 21.47, *p_*corrected*_* < 0.001, η^2^_*p*_ = 0.28], indicating that the accuracy of artificial micro-expression recognition was higher post-test than pre-test in both the first and the second visits (first visit: 81.96 ± 12.48 vs. 57.39 ± 13.79; second visit: 80.40 ± 16.53 vs. 69.46 ± 14.77). However, during these two training programs, both the main effect of stimulation [first visit: *F*(1,56) = 0.99, *p*_*corrected*_ = 0.33, η^2^_*p*_ = 0.02; second visit: *F*(1,56) = 0.01, *p*_*corrected*_ = 0.94, η^2^_*p*_ = 0.00] and stimulation × testing stage interaction [first visit: *F*(1,56) = 1.11, *p_*corrected*_* = 0.30, η^2^_*p*_ = 0.02; second visit: *F*(1,56) = 0.98, *p*_*corrected*_ = 0.33, η^2^_*p*_ = 0.17] did not reach significance.

Similar training effects were found in spontaneous micro-expression recognition (see Figure 2B). The main effect of the testing stage was significant (first visit: *F*(1,56) = 23.78, *p_*corrected*_* < 0.001, η^2^_*p*_ = 0.30, post-test accuracy: 53.35 ± 17.25 vs. pre-test accuracy: 40.71 ± 18.01; second visit: *F*(1,56) = 10.76, *p_*corrected*_* < 0.01, η^2^_*p*_ = 0.16, post-test accuracy: 55.90 ± 16.48 vs. pre-test accuracy: 47.41 ± 15.23). This noteworthy improvement was highly consistent with our observations in the artificial micro-expression recognition test, although it was much more difficult to identify a spontaneous micro-expression correctly. Furthermore, this training effect can be sustained for a significant period, as the accuracy of the pre-test in the second visit after 2 weeks was higher than the baseline created during the first visit [*t*(57) = 2.40, *p_*corrected*_* < 0.05]. The main effect of stimulation was not significant [*F*(1,56) = 2.59, *p_*corrected*_* = 0.11, η^2^_*p*_ = 0.04]. Of note, stimulation × testing stage interaction was significant in the first visit [*F*(1,56) = 4.17, *p_*corrected*_* < 0.05, η^2^_*p*_ = 0.07]. A simple effect analysis showed a higher accuracy of spontaneous micro-expression recognition for the anodal condition (*M* = 58.80, *SD* = 15.74) compared with the sham condition (*M* = 47.52, *SD* = 17.14) in the post-test (*p_*corrected*_* < 0.05). Meanwhile, there was no significant difference between the anodal (*M* = 41.11, *SD* = 18.94) and sham (*M* = 40.28, *SD* = 17.28) groups in the pre-test (*p_*corrected*_* = 0.86). We also found marginally significant interaction in the second visit [*F*(1,56) = 3.11, *p_*corrected*_* = 0.08, η^2^_*p*_ = 0.05]. The simple effect analysis showed an improvement in the accuracy of spontaneous micro-expression recognition through training in the anodal group even if no stimulation was applied in the second visit (anodal group: post-test accuracy: 59.11 ± 15.45 vs. pre-test accuracy: 46.30 ± 17.04, *p_*corrected*_* < 0.01; sham group: post-test accuracy: 52.47 ± 17.12 vs. pre-test accuracy: 48.61 ± 13.23, *p_*corrected*_* = 0.30). As all the results until this stage showed no stimulation effect from training on artificial micro-expression, we did not consider it in the next analysis.

**FIGURE 3:**
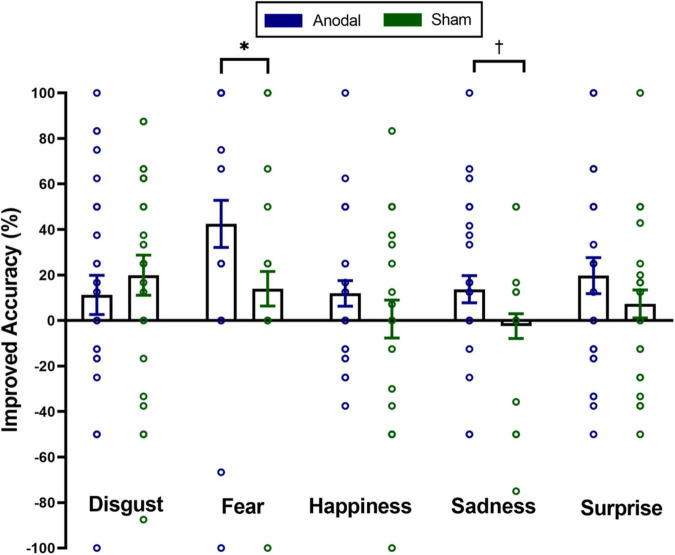
Mean improved accuracy on distinct emotions of spontaneous micro-expressions for anodal and sham group in the first visit. Comparisons of the differences of distinct emotions between the two groups showed that the anodal group improved more than the sham group on spontaneous micro-expressions of fear and sadness. The significance levels are marked by different symbols. “*” and “†” indicate *p* < 0.05 and *p* < 0.1 (two-tailed), respectively. Error bars represent standard error of mean (SEM).

To investigate the impact of tDCS on micro-expression recognition training of different emotions, we conducted a mixed two-way repeated ANOVA on the improved accuracy of the micro-expression recognition. For the first visit, the main effect of stimulation was significant, *F*(1,56) = 5.11, *p_*corrected*_* < 0.05, η^2^_*p*_ = 0.08. The main effect of emotion was marginally significant, *F*(4,53) = 2.26, *p_*corrected*_* = 0.08, η^2^_*p*_ = 0.15. Stimulation × emotion interaction was not significant, *F*(4,53) = 1.24, *p_*corrected*_* = 0.31, η^2^_*p*_ = 0.09. For the second visit, the main effect of stimulation was marginally significant, *F*(1,56) = 3.35, *p_*corrected*_* = 0.07, η^2^_*p*_ = 0.06. Neither the main effect of emotion [*F*(4,53) = 0.41, *p_*corrected*_* = 0.80, η^2^_*p*_ = 0.03] nor the interaction between stimulation and emotion [*F*(4,53) = 0.65, *p_*corrected*_* = 0.63, η^2^_*p*_ = 0.05] was significant. Furthermore, we also conducted five independent sample *t*-tests (classical frequentist statistical analysis) and Bayesian independent *t*-tests on the improved accuracy of spontaneous micro-expression recognition between two groups to analyze which emotion training effect can be improved using anodal stimulation on exploratory. The results showed the significantly higher improved accuracy in the anodal group for only the spontaneous micro-expression of fear [*t*(56) = 2.20, *p_*uncorrected*_* < 0.05, Cohen’s *d* = 0.58, BF_10_ = 1.9] and sadness [*t*(56) = 2.00, *p_*uncorrected*_* = 0.051, Cohen’s *d* = 0.525, BF_10_ = 1.4] in the first visit (see Figure 3). We found no significant difference between the two groups for the improved accuracy of spontaneous micro-expressions of disgust [*t*(56) = −0.71, *p_*uncorrected*_* = 0.48, Cohen’s *d* = −0.19, BF_10_ = 0.33], happiness [*t*(56) = 1.13, *p_*uncorrected*_* = 0.26, Cohen’s *d* = 0.30, BF_10_ = 0.46], and surprise [*t*(56) = 1.24, *p_*uncorrected*_* = 0.22, Cohen’s *d* = 0.32, BF_10_ = 0.50] in the first visit. We didn’t find similar significant results in the second visit (*ps* > 0.28, BF_10_ < 0.44).

In addition, we tested whether empathy played a role in the effect of tDCS on the improved accuracy of spontaneous micro-expression recognition. Spearman rank correlations were performed separately in the anodal and sham groups to examine the relationship between empathy traits and the improved accuracy of overall spontaneous micro-expressions and the spontaneous micro-expressions of fear and sadness. We found different relationships between the improved accuracy of fear spontaneous micro-expressions and personal distress, with a positive correlation in the anodal group (Spearman’s *rho* = 0.43, *p_*uncorrected*_* = 0.02, BF_10_ = 3.20) but no correlation in the sham group (Spearman’s *rho* = 0.13, *p_*uncorrected*_* = 0.50, BF_10_ = 0.43). There was no significant correlation for other empathy traits (empathic concern, perspective taking, and fantasy) in the two groups (*ps* > 0.11, BF_10_ < 0.88). Furthermore, no significant correlation was found between empathy traits and the improved accuracy of overall spontaneous micro-expression or spontaneous micro-expression of sadness in the anodal or sham group (*ps* > 0.16, BF_10_ < 0.43) (see Table 2).

**TABLE 2:** Correlations between empathic traits and mean improved accuracy of overall spontaneous micro-expressions, and mean improved accuracy of spontaneous micro-expression of fear and sadness in the anodal and sham group.

	Perspective taking	Fantasy	Empathy concern	Personal distress
*Anodal group*				
Mean improved accuracy of overall spontaneous micro-expression	−0.07 (0.26)	−0.002 (0.25)	−0.003 (0.21)	0.15 (0.23)
Mean improved accuracy of spontaneous fear micro-expression	−0.07 (0.23)	0.3 (0.88)	0.1 (0.28)	0.43* (3.20)
Mean improved accuracy of spontaneous sad micro-expression	−0.01 (0.24)	−0.002 (0.23)	0.01 (0.23)	−0.22 (0.43)
*Sham group*				
Mean improved accuracy of overall spontaneous micro-expression	−0.12 (0.24)	0.07 (0.22)	0.19 (0.33)	0.24 (0.41)
Mean improved accuracy of spontaneous fear micro-expression	−0.08 (0.28)	0.01 (0.26)	0.07 (0.26)	0.13 (0.43)
Mean improved accuracy of spontaneous sad micro-expression	−0.1 (0.26)	0.19 (0.38)	0.05 (0.24)	0.12 (0.29)

The results are presented as Spearman’s rho (BF10). The significance levels are marked by different symbols. “*” indicates p < 0.05 (two-tailed).

## Discussion

### Training Effect for Both Artificial and Spontaneous Micro-Expression

In the present study, we administered anodal stimulation over the rTPJ and used the Chinese version of METT to investigate the combined effect on artificial and spontaneous micro-expressions as well as the role of empathy in this process. We first expected a significant training effect for both artificial and spontaneous micro-expression using the Chinese version of METT. Results revealed that the accuracy of the participants’ detection of artificial and spontaneous micro-expressions improved after completing the training. Furthermore, the learning efficiency could be observed 2 weeks after training. Results were consistent with previous studies. Hurley’s (2012) randomly assigned participants who completed the METT detected micro-expression more accurately than those in a control group. Russell et al. (2006) determined that the ability to recognize emotional facial expressions and micro-expressions improved significantly in patients with schizophrenia using METT. Besides, concomitant changes in visual attention on facial emotion sustained and could be observed 1 week after METT training (Russell et al., 2008). Hurley et al. (2014) showed that learning efficiency could be retained 6−20 months after training. However, the learning effects could not be expected to last for such a long time in the present study, because there was only a 2-week duration between two visits in the experiment. As a whole, we believed that as a training tool, METT had a training effect not only on artificial micro-expressions but also on spontaneous micro-expressions, and this training effect could last for sustained periods.

### Enhanced Effect for Spontaneous Rather Than Artificial Micro-Expression

As hypothesized, there were stark differences in the training effect between spontaneous and artificial micro-expressions under anodal stimulation over the rTPJ. Our data suggested that the stimulation over the rTPJ was associated with an enhanced micro-expression recognition training effect, but only for spontaneous (and not artificial) micro-expressions. The artificial micro-expression in Ekman’s METT was artificially synthesized by presenting an emotional expression with both a forward and backward neutral expression. However, the spontaneous micro-expression in our study was presented via the video recording that showed the complete spontaneous process from the formation to the changes of the facial micro-expression. The former was dynamic but discrete, whereas the latter was continuous and more in line with the actual social context in which the rTPJ played an important role (Loscalzo, 2011; Tang et al., 2015). Moreover, the emotions of the expressions sandwiched between the two neutral expression pictures in the artificial micro-expressions were basic, obvious, and intense, while the emotions of spontaneous micro-expressions were suppressed and partial though also basic. The facial cues provided by artificial micro-expressions were simpler and more evident than those generated by spontaneous micro-expressions. Therefore, the recognition of artificial micro-expression was more like the response to the familiar and rapid information, in which implicit perception processing was a predominant driver (Rellecke et al., 2012). Given we focused on enhancing explicit identification ability to improve the training effect in the present study, anodal stimulation had a feeble effect. Furthermore, in contrast to artificial micro-expressions, the facial information presented by spontaneous micro-expressions was subtle and ambiguous. Therefore, successful recognition of the spontaneous micro-expressions required the higher capability to attribute a person’s intentions and emotional state based on current cues. Additionally, decades of research provide evidence that the rTPJ played a specific role in this ability, namely ToM, which incorporated the information received into a context to represent and interpret the ideas of others (Mitchell, 2008; Carter and Huettel, 2013; Mai et al., 2016). Therefore, anodal stimulation promoted the training effect of spontaneous micro-expression recognition. Above all, anodal stimulation over the rTPJ could enhance the training effect of spontaneous micro-expressions rather than artificial micro-expressions.

### Enhanced Specific Emotions Under Right Temporal Parietal Junction in People With Different Empathy Traits

We found significantly higher improved accuracy of fear spontaneous micro-expression in the anodal group than in the sham group. Fear is a visual cue associated with fast-approaching danger and requires an immediate response so that fear always attracts salient attention (Jack et al., 2014). The rTPJ engaged in attentional orienting toward threat information could contribute to the increased training effect of stimulation on the spontaneous micro-expressions of fear (Sagliano et al., 2019). This finding was consistent with previous research. Donaldson et al. (2019) found that the rTPJ anodal HD-tDCS could influence fear facial emotion recognition. Besides, they suggested that the rTPJ involvement in facial emotion processing depended on the intensity and salience/valence (negativity/threat) of the emotion. Surprisingly, we also found an increased training effect from anodal stimulation over the rTPJ for the spontaneous micro-expression of sadness. When recognizing expressions of sadness, eye cues had greater significance than other cues and tended to capture most attentional resources (Eisenbarth and Alpers, 2011). rTPJ was proved to be linked to eye gaze and sensitive to eye information (Kelley et al., 2021), and might therefore lead to an improved effect of training on spontaneous micro-expressions of sadness.

We also explored the role of empathy in the effect of tDCS over the rTPJ on micro-expression training. We found different relationships between the improved accuracy of spontaneous micro-expressions of fear and personal distress, with a positive correlation in the anodal group but no correlation in the sham group. Personal distress is an index of the individual’s own feelings of fear, apprehension, and discomfort when they witness the negative experiences of others. Personal distress may be positively associated with sensitivity to dangerous signals, but it is relatively more self-focused that it does not contribute to and is even adverse to emotional reaction (Davis, 1980, 1983). Therefore, personal distress does not influence the training effect in the sham group. Based on the development pattern of empathic tendencies set forth by Hoffman, personal distress seems to be an automatic imitation of the state observed in those who suffer from a disaster. It appears to be the preliminary empathy phase in early development, a phase in which children cannot differentiate between themselves and others effectively. As self-centered empathic distress is transformed into other-oriented concern, the ability to apprehend others’ mental and emotional states develops (Hoffman, 1976). Existing neuroimaging findings suggested that the rTPJ region played a crucial role in the mechanism that enabled the control of automatic imitative responses and allowed representation of the self to be inhibited and that of others to be enhanced (Brass et al., 2009; Spengler et al., 2009). Furthermore, causal evidence for the role of the rTPJ in self-other control was derived from studies measuring the effects of magnetic or electric stimulation over this area. Repetitive transcranial magnetic stimulation (rTMS) targeted to the rTPJ has been shown to be involved in switching between self and other representations (Sowden and Catmur, 2015). Additionally, anodal tDCS over the rTPJ could inhibit self-centered concern and promote other-oriented concern, facilitating the transformation of self to other representations (Sowden et al., 2015). Therefore, in the current study, the anodal stimulation over the rTPJ suppressed self-centered empathic distress and promoted other-oriented concern to improve the ability to read the emotional states of others, which was embodied in the enhanced effect of training on fear spontaneous micro-expressions.

### Limitations and Future Directions

This study has several limitations and there are some possible directions for further study. One limitation was that emotional dimensions between artificial and spontaneous micro-expression stimuli were inconsistent. Spontaneous micro-expressions of anger were lacking compared to artificial micro-expressions because of unsatisfactory labels and the difficulty in eliciting the emotion in a laboratory context (Yan et al., 2014). Furthermore, although the study was motivated based on the specific role of the rTPJ in emotional recognition, it couldn’t be denied that the anodal stimulation activated other brain regions that might play a similar or synergistic role. Therefore, further work is required to establish a more refined neural mechanism of micro-expression training using tDCS combined with fMRI. Besides, in the current study, we applied anodal stimulation over the rTPJ that played an important role in the explicit process of facial emotion recognition to enhance micro-expression training effect. However, an issue that was not addressed in this study was whether micro-expression recognition could be improved by manipulating the implicit perception process of facial emotion recognition. Micro-expressions are presented as brief durations, so judgments are thought to rely in part on intuitive, biologically based emotion processing. Therefore, the causal relationship between perception and emotional recognition is also in need of further investigation (Svetieva and Frank, 2016). In addition, other individual characteristics, such as “the Big Five,” that may contribute to modifying the effect of tDCS over the right TPJ on micro-expression training, are a worthy focus for future studies. Moreover, in a meta-analysis across studies including populations characterized as psychopathic, abusive, conduct-disordered, aggressive, unsocialized, criminal and antisocial traits/behaviors were most consistently associated with deficits in recognizing the facial expression of fear. In a future study, we plan to explore the training effect of the combined use of tDCS over the rTPJ and the Chinese version of METT in clinical populations.

## Conclusion

This study provided convincing evidence that METT can improve both artificial and spontaneous micro-expression recognition. Anodal stimulation over the rTPJ was associated with an enhanced micro-expression recognition training effect, particularly for spontaneous micro-expressions related to fear. The improved accuracy of recognizing fear spontaneous micro-expressions was positively correlated with personal distress in the anodal group but not in the sham group. The results are promising but preliminary. The combined use of tDCS and METT can be a promising method for teaching and enhancing micro-expression recognition.

## Data Availability Statement

The original contributions presented in this study are included in the article/supplementary material, further inquiries can be directed to the corresponding author/s.

## Ethics Statement

The studies involving human participants were reviewed and approved by the Institutional Review Board of the State Key Laboratory of Cognitive Neuroscience and Learning at Beijing Normal University. The patients/participants provided their written informed consent to participate in this study. Written informed consent was obtained from the individual(s) for the publication of any potentially identifiable images or data included in this article.

## Author Contributions

CL conceived the study and acquired the funding. RS, ZL, JL, and ST conducted the experiments with the help of YG and XS. RS analyzed the data with critical comments from CL. RS and YG wrote the manuscript with help from CL, HW, and XS. All authors helped to refine the final experimental design and commented on the manuscript.

## Conflict of Interest

The authors declare that the research was conducted in the absence of any commercial or financial relationships that could be construed as a potential conflict of interest.

## Publisher’s Note

All claims expressed in this article are solely those of the authors and do not necessarily represent those of their affiliated organizations, or those of the publisher, the editors and the reviewers. Any product that may be evaluated in this article, or claim that may be made by its manufacturer, is not guaranteed or endorsed by the publisher.
